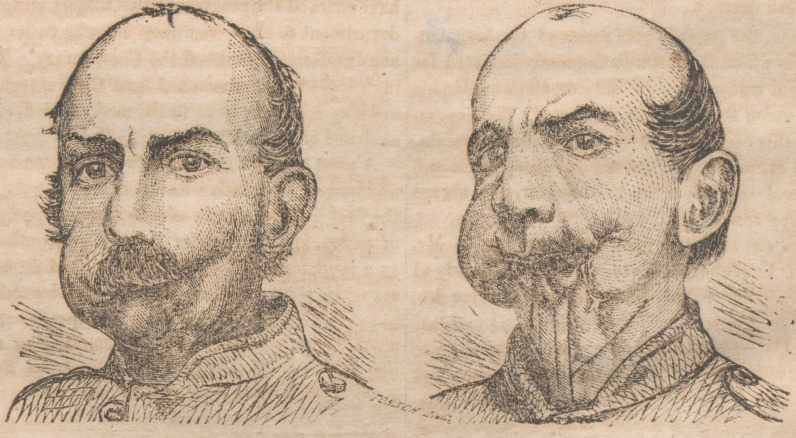# Operations in Reparative Surgery

**Published:** 1864-07

**Authors:** Chas. Bell Gibson

**Affiliations:** Surgeon P. A. C. S.


Art. V.—*Operations in Reparative Surgery.* By Chas. Bell Gibson, Surgeon P. A. C. S.

W. M. Wyatt, private, Page's battery, 1st regiment Virginia artillery; age 46; occupation a farmer; was admitted into General hospital, No. 1, Richmond, Sept. 17, 1863. Wounded 13th September in the face by a piece of shell. His condition, on entering the hospital, was as follows:

The lower jaw was fractured and carried away from the first molar tooth of the right side, near the angle of the jaw on the left side; the tongue was badly lacerated, and the soft tissues forming a portion of the cheeks, the whole lower lip, and the original covering of the chin had been carried away.

His appearance was frightful and most pitiable, and as sloughing had commenced, the prognosis was most unfavorable.

Fortunately, however, in a few days the sloughing process ceased, and although suppuration was profuse and very offensive, the granulating process at last became fairly established, and by the 10th November (in fifty-four days after receipt of injury) he was well enough to go to his home on furlough. He had been told that an attempt might be made to improve his appearance by an operation at a future day, and he accordingly returned to the hospital early in January.

By this time cicatrization had occurred. Irregular and lumpy cicatrices extended into the cheeks, from the corners of the upper lip (which had not been involved in the wound) and down upon the throat; and the tongue appeared in the chasm representing his mouth, adhering to the transverse edge of the cicatrix two inches below the border of the upper lip.

After careful inspection as to the best means of relieving the deformity, it was determined to attempt to make a new lower lip, by dissecting up the tissues of the throat and cheeks, sliding them to a level with the border of the upper lip, and securing them in position by sutures.

The operation was performed on the 10th of January, 1864, without chloroform, as it was desirable the patient should not incur the danger of blood passing into the air passages; and the extreme suffering, necessarily attendant, was borne with patience and courage rarely witnessed.

An incision three inches long was made downwards in the centre of the tissues of the throat, terminating about the middle of the thyroid cartilage. From the termination of this incision another was carried, first on the right side and then on the left, upwards and backwards towards either angle of the jaws, each to the extent of three inches; and thus two flaps were marked out. These flaps were then dissected

(about one-fourth of an inch in thickness) from the sub-jacent tissues; so that, when the dissection was completed, the two flaps, each being seized at the central incision, could be raised and brought up so as to present an opposing margin or surface to the upper lip. As had been anticipated, it was found that the flaps now required to be incised so as to prevent the edges by which they were to be united to each other in the centre from overlapping; and accordingly about a quarter of an inch in width was removed from each flap along the edges. Then, being again brought up to the border of the upper lip, the flaps were united, in a central line, by interrupted sutures of silver wire. Two incisions were then made from either angle of the new mouth outwards, and the lumpy and unsightly cicatrices, before mentioned, were cut out, and the wounds also united by silver wire. Adhesive strips and a bandage completed the operation. The lateral dissection of these flaps, of course, left a considerable space on the throat and neck of raw surface. This was expected to granulate and cicatrize, in due time, under water dressing. Directions were given to very careful attendants as to diet and drinks, and an anodyne administered.

On the fourth day the parts were examined, and with the exception of the suture at the upper end of the central line, which had ulcerated through, all was doing well; perfect union had occurred. It was deemed best, however, as the sutures were not producing irritation, to leave them; and thus they were not removed till the tenth day. At this time the parts were perfectly consolidated; the flaps not only united in the centre, but adherent to the subjacent surface. The wound below was suppurating and granulating kindly.

On the 1st of March the patient left the hospital again, for his home, greatly improved in appearance and in his power of articulation. It is probablethat some contrivance may be adopted by which he will one day be able to masticate his food.

It is regretted that the record of a case has been lost, in which the upper lip and nostrils were extensively mutilated by a portion of shell, and in which plastic surgery was also made eminently useful. The operation was performed in General hospital, No. 1, in the fall of 1863, upon a patient named Cook, from Georgia. He was returned to his regiment, and when last heard from was on duty with it.

*Case Second.*

Private R. A. Green, company "C," 18th Virginia regiment, was sent to the reporter by Surgeon Wm. A. Carrington, Medical Director, for examination. He was found to have epithelial cancer of the lower lip, extending from the right commissure to half an inch beyond the middle line, and downwards towards the chin about an inch.

In view of the probable lengthening of life, and possible eradication of the disease by operative procedure, it was advised that the tumour should be removed. The patient was accordingly admitted into General hospital No. 9, and the operation performed on the 29th of May, 1864.

Owing to the extent of the disease, it was deemed impossible to use, effectually, the simple operation of the V-incision, and it was determined to raise and transplant a flap to take the place of the diseased mass and to form a new lip.

A very small amount of chloroform being given, an incision was made through the right commissure of the lips, to the extent of half an inch; and from this incision another, vertical one, to the extent of an inch and a quarter. A similar straight incision was then made near the left commissure, and the two lower ends of these incisions were connected by a transverse incision. In this way nearly the whole lip was removed, including the diseased portion and a quarter of an inch of healthy tissues around it. The two straight incisions were then continued two inches down over the throat, and the flap dissected and raised between them. This flap was then seized and (the head being slightly bent forwards) brought up to oppose the upper lip, and to meet the incision *at* the right and *near* the left commissure—this covering over the space from which the diseased tissue had been removed. The flap was found to fit accurately, and silver-wire sutures were employed. Adhesive strips, a compress and a bandage were applied. On the 30th the patient was transferred to Chimborazo hospital, and entered ward "E," 5th division.

On the 9th June, the sutures were removed by Assistant-Surgeon Cherry (to whom the operator is greatly indebted for his kind and skillful management of the case while under his care) and perfect union found to have occurred. The operation performed in this case is, it is believed, of French origin, and is termed the operation by "Glissement" or "Sliding," and has been several times done by the reporter, and always with the same happy result.

One of the cases was operated upon ten years ago, (a Mr. Smith, formerly of Lunenburg county, and more recently of Ashland, Hanover county, in this State) and in it there has been no return of the disease.

Another case, Mr. ——, of Hardy county, Virginia, was operated on in the summer of 1862, at Coyner's Springs, and a few months ago had shown no return of the disease.

In other cases the reporter is unable to say anything as to the return or non-return of the disease.

The common opinion of the epithelial cancers being trivial diseases in comparison with the schirous and medullary, has been clearly proved by M. Paget to be incorrect. The average duration of life with them is, probably, less than four years, and no great prolongation of life would appear, by cases that have been collected, to be obtained by their removal; but it is respectfully submitted whether the prospect of recovery is not improved by an operation in which a perfectly sound and healthy tissue is made to cover a space from which not only the diseased, but a considerable portion of sound tissue, bordering on the diseased, has been removed.

C. S. Medical & Surgical Journal.

## Figures and Tables

**Figure f1:**